# Supplementary material for: A new method for the study of biophysical and morphological parameters in 3D cell cultures: Evaluation in LoVo spheroids treated with crizotinib
Source: PLoS One. 2021 Jun 8;16(6):e0252907. doi: 10.1371/journal.pone.0252907 (PMC8186796; doi:10.1371/journal.pone.0252907)
Supplement: S1 Dataset — Measurements raw data of mass density, weight, and diameter of live and fix LoVo spheroids treated with crizotinib 250 and 500 nM. (PDF) [file pone.0252907.s002.pdf]

Measurements raw data of mass density, weight, and diameter of live and fix LoVo spheroids treated with crizotinib 250 and 500 nM

LIVE CTRL

Density AVG [kg/m3]	Density STD [kg/m3]	Weight AVG [ng]	Weight STD [ng]	Diameter AVG [μm]	Diameter STD [μm]
1018.2	3.4	4586	1525	203	22
1016.4	2.3	3842	1090	192	18
1017.9	3.0	4278	1495	198	23
1016.8	0.7	8152	842	248	9
1016.6	1.5	8714	1187	253	12
1017.8	3.6	7714	2299	241	28
1016.6	3.3	9807	2861	261	30
1018.6	3.8	4570	2169	200	33
1018.0	3.8	5124	2510	208	32
1016.1	1.0	7468	812	241	9
1016.2	1.6	6807	757	234	9
1018.1	1.2	7606	753	242	8
1017.5	1.3	4833	736	208	11

LIVE 250 nM

Density AVG [kg/m3]	Density STD [kg/m3]	Weight AVG [ng]	Weight STD [ng]	Diameter AVG [μm]	Diameter STD [μm]
1020.7	2.5	5665	1274	218	17
1021.6	1.9	5787	1000	221	12
1020.2	0.9	4053	283	196	5
1021.6	0.4	4272	390	200	6
1028.1	4.6	1630	530	143	15
1018.6	1.2	3332	301	184	6
1024.3	1.2	4956	331	210	5
1023.6	1.4	2441	282	166	6
1024.3	2.7	2831	483	174	10
1020.8	1.8	3058	381	179	8
1020.3	1.4	2898	246	176	5
1021.7	0.7	2228	128	161	3
1021.5	1.9	2819	276	174	6
1021.7	1.6	2189	249	160	6
1027.9	2.6	3181	117	181	2

LIVE 500 nM

Density AVG [kg/m3]	Density STD [kg/m3]	Weight AVG [ng]	Weight STD [ng]	Diameter AVG [μm]	Diameter STD [μm]
1019.5	0.6	3593	205	189	4
1017.5	1.4	3912	303	194	5
1025.2	1.0	2598	204	169	4
1023.8	0.6	2645	140	170	3
1018.2	1.2	3540	411	188	7

1028.6	1.6	2699	118	171	3
1029.7	1.4	2941	186	176	4
1020.6	2.1	3033	536	178	10
1029.5	1.1	2579	180	168	4
1027.7	1.9	2425	292	165	7
1016.3	2.0	3915	886	194	14

FIX CTRL

Density AVG [kg/m3]	Density STD [kg/m3]	Weight AVG [ng]	Weight STD [ng]	Diameter AVG [μm]	Diameter STD [μm]
1015.4	0.9	4945	566	210	8
1017.5	0.9	8529	586	252	6
1020.3	1.5	5787	701	221	9
1016.1	0.8	7031	304	236	3
1017.1	1.9	6690	1187	232	15
1019.5	1.1	5218	517	214	7
1015.5	0.9	6978	296	236	3
1019.8	1.5	5401	748	216	10
1018.2	1.0	8074	880	247	9
1017.1	0.8	6149	389	226	5
1017.6	0.5	7493	405	241	5
1020.1	2.2	6181	1159	225	14
1018.1	0.9	4116	341	198	6
1014.5	0.6	6619	549	232	6
1018.8	1.1	6582	551	231	6
1017.5	1.8	5815	752	221	10

FIX 250 nM

Density AVG [kg/m3]	Density STD [kg/m3]	Weight AVG [ng]	Weight STD [ng]	Diameter AVG [μm]	Diameter STD [μm]
1025.8	1.0	2933	202	176	4
1030.2	0.8	2368	178	164	4
1026.0	1.5	2446	238	166	5
1029.1	0.6	2501	106	167	2
1026.9	0.6	2793	86	173	2
1030.2	1.4	2505	203	167	5
1028.4	1.1	2886	304	175	6
1028.8	0.8	2797	192	173	4
1024.8	0.9	3260	229	182	4
1028.4	0.8	3295	132	183	3
1027.6	0.9	2949	189	176	4
1029.3	1.4	3344	326	184	6
1028.1	0.3	2564	104	168	2
1027.4	1.1	3231	203	182	4
1027.7	0.6	3308	152	183	3
1029.3	2.0	2402	169	164	4

FIX 500 nM

Density AVG [kg/m3]	Density STD [kg/m3]	Weight AVG [ng]	Weight STD [ng]	Diameter AVG [μm]	Diameter STD [μm]
1026.6	0.8	2567	134	168	3
1026.0	0.6	2720	141	172	3
1027.6	0.8	2481	98	166	2
1029.9	1.3	2461	204	166	5
1032.4	2.0	2253	207	161	5
1031.5	1.1	2367	95	164	2
1030.9	1.3	2386	144	164	3
1030.1	1.3	2496	173	167	4
1029.0	0.3	2116	44	158	1
1028.4	2.7	1883	321	151	8
