# Supplementary material for: A new method for the study of biophysical and morphological parameters in 3D cell cultures: Evaluation in LoVo spheroids treated with crizotinib
Source: PLoS One. 2021 Jun 8;16(6):e0252907. doi: 10.1371/journal.pone.0252907 (PMC8186796; doi:10.1371/journal.pone.0252907)
Supplement: Attachment — Submitted filename: Review_Weiren Huang.doc [file pone.0252907.s003.doc]

**Review**

The manuscript involves a 3D culture system to study the biophysical and morphological parameters in LoVo spheroids treated with TKI. The authors used a new fluidic-based measurement, combined with confocal imaging and protein quantification methods, to show that they can provide robust phenotypical characterization of the spheroids structure, offering insights on the spheroids bulk and an accurate measurement of the tumor density. They also represented that this analysis helps overcome the technical limits of the imaging that it is difficult to penetrate the thickness of 3D structures. More importantly, they demonstrated that TKI treatment has an impact on mass density, which represents a key marker of cancer cell treatment. Overall, the accurate characterization of in vitro spheroid models is of great significance for cancer research and clinical treatment. The experiments described here are well designed but there are some concerns that require additional analysis and/or discussion.

1. I wonder if the W8 fluidic system described in this study is applicable to some other cell types? If yes, please point out the relevant evidence. If no, please explain the reason.

2. Materials and Methods:

a. Line 115, the reference “Cristaldi et al.” should be cited with the number [22] in the text.

b. Line 121, a brief description of the NanoOrange methods should be provided.

3. Results and Discussion

a. Bright field images of spheroid cultures in the presence or absence of TKI should be provided, so that we can observe their morphological characteristics during culture.

b. Line 177-178 “This is in line with an antiproliferative activity of the inhibitor”, please add a citation source.

4. Figure 3B:

a. All the fluorescent images provided only show single spheroid. Please provide a more comprehensive view at lower magnification to capture more spheroids.

b. The authors do not show how many spheroids were analyzed in each group, and how many times were these experiments repeated? In addition to showing representative fluorescent images, the authors need to present these results with statistical analysis.

5. Patient-derived organoid models are thought to be the next generation in vitro models that have been shown to closely recapitulate patient's disease. Unlike cell lines, organoids are both genetically and phenotypically more stable and recapitulate the main features of patient’s tumor tissues. In terms of preclinical studies, organoid may be more suitable for drug screening and tumor biology research than spheroids derived from self-organization of cancer cell lines. Please discuss this point. Thank you.
